# Oral health literacy among migrant mothers in Sweden. A qualitative study

**DOI:** 10.1080/00016357.2023.2291206

**Published:** 2024-03-26

**Authors:** Elena Shmarina, Malin Stensson, SANT-Network, Brittmarie Jacobsson

**Affiliations:** a Department of Oral Diagnostics, Faculty of Odontology, Malmö University, Malmö, Sweden; b Kalmar County Council, Public Dental Service, Oskarshamn, Sweden; c Centre for Oral Health, School of Health and Welfare, Jönköping University, Jönköping, Sweden; d CHILD Research Group, SIDR, Jönköping University, Jönköping, Sweden

**Keywords:** Asylum seekers, dental health, information seeking behaviour, oral health information, refugees

## Abstract

**Objective:**

This interview study explored the oral health literacy of migrant mothers in Sweden, with special reference to where and why they access information about oral health and how they determine the credibility of such information.

**Material and method:**

In-depth interviews were conducted with seven migrant mothers of children up to 10 years old. The mothers had entered Sweden from 2015 onwards and had been resettled in Kalmar County, Sweden. Their native language was Somalian, Dari or Arabic. The interview questions concerned the participants’ experiences of seeking oral health information, as well as oral health in general and dental health services. The interviews were analysed by qualitative content analysis.

**Findings:**

The main findings indicate that migrant mothers used information sourcing as a pathway to solve oral health literacy tasks. Three main categories were identified, each with subcategories, describing the migrant mothers’ experiences of accessing and evaluating oral health information: *‘accessible source of information’, ‘needs-related purpose of seeking information’ and ‘trustworthiness assessment’*. The migrant mothers reported that in case of a dental emergency or general queries, they sought oral health information from professionals and social sources. Moreover, they perceived oral health information to be most reliable when it was provided by dental professionals, was a recurring theme, or constituted majority opinion.

**Conclusion:**

To improve oral health literacy in migrant mothers of young children, it is important not only to provide consistent and recurrent oral health information through accessible information channels, but also to adapt dental care to be more culturally appropriate.

## Introduction

Oral health literacy (OHL) has been identified as crucial determinant of oral health promotion and prevention of oral diseases [1]. Drawing on a definition of health literacy, OHL is defined as the degree to which the individuals have the capacity to obtain, process, and understand basic oral health information and services needed to make appropriate oral health decisions [1]. This is a relatively new field of research, only recently acknowledged by the scientific community [2]. Interest in OHL is driven by disparities in oral health, particularly with reference to disadvantaged groups, including migrant populations [1].

Research data from different countries reveal that children of migrant background have significantly poorer oral health than their non-migrant peers. For example, a scoping review of North American research revealed that the oral health of children of newly arrived migrants was poorer than that of their American counterparts [3]. In a Danish study, mean caries experience was three to four times higher in pre-school children of migrant mothers than among children of Danish-born mothers and mean caries experience in teenagers was twice as high in those with migrant mothers [4]. In accordance with international findings, Swedish data show that migrant background is strongly associated with dental caries experience in pre-school children [5,6].

It has been argued that multiple factors influence the development of dental caries and oral health problems in migrant children, including country of birth, cultural influences, as well as the parents’ OHL. Parents’ OHL alone, and in combination with cultural influences, plays an important role in determining caries development in the children: it influences diet, self-care and access to dental services [7–9]. Therefore, fundamental to this study is the tenet that in order to promote oral health in the children of migrants, we first need to explore migrant parents’ OHL, to identify obstacles to promotion of oral health and prevention of oral diseases in their children.

The rapidly growing number of migrants to Sweden in the past decade adds another level of complexity to improving oral health outcomes in the children of migrants. Dental services personnel now encounter much more language, cultural, religious and ethnic diversity. A cross-sectional study mapping the health of newly arrived migrants in Sweden showed that nearly 65 per cent face health literacy challenges [10], which might greatly affect their ability to apply health information to make decisions about their children’s health. Nevertheless, the role of families and parents in particular is of great importance for achieving positive oral health outcomes in their children. A systematic review of the literature disclosed an association between low parental OHL and dental caries in children [11]. It has been found that children whose parents or caregivers had lower levels of OHL were more likely to have dental caries. In some studies, included in the systematic review, this result remained significant even after controlling for confounders. According to the authors, a possible explanation for this trend was that parents with lower OHL may have inadequate knowledge of how to prevent dental caries in their children, or have more difficulty in understanding oral health information [11]. Today, information related to oral health is disseminated through all media sources, including social media and various websites on the Internet and can readily be accessed by parents. However, the quality and reliability of information resources can vary. Hence the parents must be capable of critical examination of the material. Moreover, in order to ensure optimal care of these children, dental health care professionals need to take into account the parental level of health literacy.

## Aim

The overall aim of this study was to explore the oral health literacy of migrant parents, with special reference to their capacity to access and utilize oral health information to maintain and promote oral health in their children.

The following specific research questions were addressed:

- Where do migrant parents source information on oral health?
- Why do migrant parents seek information about oral health?
- How do migrant parents determine the credibility of oral health information?

## Methods

This explorative study used in-depth interviews for data collection and a qualitative content analysis method for data analysis [12]. The study was conducted in partnership between the SANT-network (The Asylum Seekers and Newly Arrived Dental Care Network of Småland, Sweden), Jönköping University and the Public Dental Service in Kronoberg and Kalmar Counties. The study was conducted in Kalmar County, Sweden. The Consolidated Criteria for Reporting Qualitative research (COREQ) was used as a reporting guideline [13].

During the planning phase, in order to enhance our understanding of migrant families’ oral health issues, public dental care coordinators for asylum seekers and newly arrived migrants were interviewed. In the context of this study, the public dental care coordinators were dental professionals who worked in particular with dental care for asylum seekers and newly arrived migrants. The cultural interpreters representing each language community, Somali, Dari and Arabic, were also interviewed to develop an understanding of cultural influence on oral health. In the present context, the cultural interpreters were migrants who had lived in Sweden for a long time, spoke Swedish fluently, and had educational and work experience in the field of dental health from their country of birth and/or from Sweden. The purpose of the interviews with public dental care coordinators and cultural interpreters was solely to enhance our understanding of the focus of this study.

A common principle for determining adequate sample size in qualitative studies is that the sample size should be sufficiently large and varied to elucidate the aim of the study [14]. To follow this principle, we applied the *Information power model* in the planning and during data collection [15]. The model suggests that sample size in qualitative studies is determined by the amount of information that the sample holds relevant to the actual study rather than the number of participants and depends on study aim, sample specificity, use of established theory, quality of dialogue, and analysis strategy. In other words, the higher the information power, the lower the number of informants needed and vice versa.

### Participants and recruitment

The sampling was purposive. The participants comprised migrant mothers resettled in Kalmar County, Sweden. The inclusion criterion was that participants should be parents of children up to ten years old. They had entered the country in 2015 at the earliest. Their mother tongue was Somali, Dari or Arabic, and they were willing to share their experiences of oral health and dental care. At the time the study was being planned, Swedish statistics reported that Somali, Dari and Arabic were the most common languages spoken by newly arrived immigrants (Source: Statistics Sweden).

Recruitment of participants was through invitation by the Public Dental Service and the Child Health Care Centre coordinators in Kalmar County, as well as through local cultural agencies and family centres. Participants were identified, contacted, recruited and interviewed consecutively until saturation had been reached with reference to the focus of interest. No further interviews were conducted once the newly collected data tended to repeat the data already collected [16].

The participants comprised seven mothers, ranging in age from 21 to 37 years (median 30 years). Five came from Syria, one from Afghanistan and one from Somalia. They spoke Arabic, Dari and Somali, respectively. On average, there were two children per mother: the range was from one to six children. The children’s ages ranged from 6 months to 16 years (median 6 years). Six of a total fifteen children to five mothers had been born after the mothers entered Sweden. The participants’ educational level varied. One mother was illiterate, two had primary education, three mothers had upper secondary school education and one had tertiary education. At the time of interview, the participants had lived in Sweden for an average of approximately six years. None had work in the host country. Two mothers had work experience from their home countries.

### Data collection

The in-depth interviews were conducted in Swedish and professional interpreters were hired when needed. Three interpreters were used in three cases, one for each language community. The interpreters were recruited through an interpreter agency used by the public dental service and had professional qualifications. Prior to the interviews, instructions were given to the interpreters.

The questions addressed various aspects of the participants’ practices in relation to seeking oral health information as well as their use of dental services, both before and after resettlement. A basic interview guide based on the research questions was used. The research questions were developed in collaboration with the SANT-network and tested in the dental care context on migrant parents with different ethnicity and backgrounds, to examine the feasibility of our approach. Each interview started by asking the participants about their experience of oral health. They were then invited to describe specific information-seeking practices, such as the use of different information sources. The participants’ answers were followed up with clarifying and exploratory questions, such as, ‘Could you tell me more?’ or ‘How did it feel?’ The participants were also encouraged to talk about and reflect on situations relevant to oral health during their lifetime, particularly their childhood.

The interviews were conducted by the first author over a period of 5 months, from August to December 2021 and ranged in duration from 40 to 65 min (mean 49 min). Recruitment and interviewing were carried out consecutively until data saturation was reached. No further interviews were conducted once the newly collected data tended to repeat the data already collected [16]. The interviews were held in a conference room in the public dental services’ premises outside working hours. All interviews were audio-recorded and transcribed verbatim, including non-verbal expressions such as pauses, laughter and sighs. All audio recordings included the Swedish interpretations of the interviews. The subsequent transcriptions included only the Swedish-language components.

### Data analysis

The transcribed data were organized and processed in NVIVO (Release 1.5.1) software, and analysed using the inductive approach for qualitative content analysis as described by Graneheim and Lundman (2004) [12].

In this study, we not only described the manifest content but also interpreted the latent content. The analysis was conducted in several steps. Firstly, the transcripts were read through several times to obtain a sense of the whole and to identify the meaning units dealing with sources of oral health information accessed by the participants, their reasons for seeking such information and also their assessment of the credibility of the information obtained. A meaning unit comprises several words, sentences or paragraphs related to each other through their content and context [12]. Next, each meaning unit was condensed in such a way that it did not lose its content and was labelled with a code. The codes were compared for differences and similarities: those with similar content were developed into subcategories and categories. Thereafter, the underlying meaning, the latent content of the categories was read, critically analysed, and formulated into a theme. All codes, categories and theme were derived from the data. Trustworthiness was ensured by an ongoing process of reflection and discussion among the authors. Throughout the analysis process, the codes, sub-categories, categories and the theme were discussed in the research group until consensus was achieved.

Direct quotations from the interviews are included in the findings section in order to illustrate how the interpretation is grounded in the data. The quotations were translated from Swedish into English by a bilingual, dentally qualified professional translator.

### Ethics

This study was approved by the Swedish Ethical Review Authority, Dnr. 2018/3674-51. The participants received written and verbal information about the aim of the study and were informed that they had the right to withdraw without having to specify the reason and that confidentiality was guaranteed. All participants signed the informed consent form.

## Findings

A theme *‘information sourcing as a pathway to solving oral health literacy tasks’* emerged in the analysis of data from interviews intended to explore OHL in a purposive sample of migrant parents. The theme implies the course of action behind the participants’ efforts to solve or answer specified oral health related issues. In this study, the process of information sourcing implies mothers’ finding and using oral health-related information to make appropriate decisions and achieve specified oral health results for their children. The participants utilized multiple information sources in different situational contexts for different purposes and sincerely trusted the source of information they primarily used. They placed value on the source of oral health information and invested effort in critical assessment of the information being presented to them.

Three main categories, *accessible source of information, needs-related purpose of seeking information and assessment of trustworthiness,* emerged in the data. The presentation of findings is organized according to the three research questions. Table 1 summarizes the formulated theme, categories and the associated sub-categories which emerged from the analysis.

**Table 1:** Overview of findings.

Research questions	Subcategories	Categories	Themes
Where do migrant parents obtain information related to oral health?	Family	Accessible source of information	Information sourcing as a pathway to solving oral health literacy tasks
	Dental professionals		
	Internet		
	Social context		
Why do migrant parents seek for information related to oral health?	Dental emergency	Need-related purpose of seeking information	
	Need for clarification		
	Teaching tool		
How do migrant parents determine credibility of information related to oral health?	Professional information	Trustworthiness assessment	
	Majority opinion		
	Recurring information		

### Accessible source of information

This category describes the sources that the participants use to obtain oral health information. The findings revealed that accessible sources were primarily used. The choice of source, whether human or electronic, depended on situational context, purpose, and accessibility, and changed with the nature of information need. The category includes four sub-categories: *family, dental professionals, social context and internet*.

#### Family

There was frequent emphasis on the importance of family as a source of oral health information. Several participants recalled receiving information from their parents, siblings or the family’s elders, such as this woman:

‘Our parents and grandparents and all of them, that was what we believed, what they told us’

A woman recalled that when she was a child, her parents kept telling her that she had to take care of her teeth and brush them to avoid future caries and dental visits. Another recalled her sister saying that she had to brush her teeth before bed. A third woman stated:

‘Eat less sweet food and then you won’t get toothache, said our parents’

Several participants talked about toothbrushing in terms of observational learning. A woman described watching her parents’ toothbrushing and then copying them. Another said:

‘We have learnt this from our parents because we were given toothbrushes by them, to brush our teeth and look after our teeth’

In case of general questions, advice from the family’s elders was followed. A woman recalled that her newly erupted permanent teeth were yellowish. The elders looked and advised:

‘They said: If you haven’t the money or you can’t find toothpaste, use salt, until I got older, and then when I got married and my financial situation improved a little, I bought such toothpaste’

Some women appreciated their husband’s competence. One of the mothers talked in terms of control of a situation. She relied on her husband who knew what to do and how to obtain the information needed. She said:

‘It is my husband who deals with that sort of thing. He can google. He can ring the medical clinic or the public dental clinic’

Another demonstrated total trust in her husband’s knowledge regarding what was the best for their children. She said:

‘Papa says, it’s better to eat honey and dates than the other stuff. That is why. Papa says it is good’

#### Dental professionals

During the interviews there was often emphasis on dental professionals as an important source of oral health information. Some mothers described actively seeking information by contacting a dental practice, while others talked about being invited to visit a dental practice with their children for a routine check-up and consequent information, such as this mother:

‘They taught us how we should brush our teeth, what we should use, which toothpaste, which we should use for his (the child’s) teeth. They gave us a book. They were really nice’

Some participants viewed dental professionals as an adequate source of information. A woman stated that a dental professional is an adequate source of oral health information if you listen carefully. She said:

‘If you listen you can get it all’

Another spoke in terms of reliability:

‘When it comes to health, the doctor is better because they know what you should do, but for other things, you talk to your parents or to your husband and so on, but not when it comes to health’

Several participants mentioned using 1177, which is the national hub for healthcare information and services in Sweden. They described contacting 1177 for advice or information on various issues, both by phone and on the web. A woman said:

‘I ring 1177 just to ask them about something I am worried about, I will ring 1177 directly and ask: Is that normal or do I need to go to the doctor?’

The participants described different ways of obtaining professional oral health information. Some preferred to receive oral information or printed materials, while others preferred visual information, such as this mother who described the great impact of a health professional who lectured on oral health:

‘She has got through to me by using pictures, a few words of text but mainly with pictures. Somehow I can take in and remember the pictures better than the text”

Moreover, the interview data also reveal that some mothers preferred to await information from dental professionals rather than to seek it actively, such as this mother:

‘I have to listen first which toothpaste is suitable, which brush is suitable. I must get advice. I can’t just start running around. Everything has to go the right way’

#### Internet

The majority of participants described Google as an important source of information. A woman stated that there are many various sources of information on oral health, but she preferred to use Google in the first place because all the information needed was available there. Another talked in terms of common activity. She said:

‘Everyone googles these days. You just have to google. You google and look. You get all the information you need’

The participants mentioned using various languages to obtain oral health information on the Internet. The choice of language depended on their proficiency in a particular language as well as on the urgency and the availability of the information being sought. Initially, they used their native language. If they could not find the information they needed in their native language or they were not satisfied with the information provided, they used the language of the host country on the internet, in this instance Swedish.

#### Social context

The participants talked about the people and the context which influenced their access to oral health information: their peers, neighbours, native Swedish speakers and school. One informant described the sources and the content of information she received growing up in her home country:

‘When I was little I heard a lot from my schoolmates and neighbours, that you shouldn’t eat a lot of chocolate and with too many sweets your teeth go bad. You lose. It happens quickly with your teeth’

Some stated that if in the host country they found themselves unable to find the information they needed, they would turn to native Swedish speakers:

‘I usually ask my Swedish friend if something happens’

Several participants referred to the information they had received at school in their home country. They described a teacher or a visiting health professional advising them not to eat sugar and instructing them in toothbrushing. One informant said:

‘We knew from school that we had to brush our teeth’

However, some women stated that the oral health information they received at school was limited to advice on toothbrushing, but with no detailed information as to what would happen to their teeth and their body if they did not brush their teeth.

Various beliefs about oral health were often mentioned. These beliefs have their origins in the participants’ cultures.

A mother assumed that eggs and milk help her baby’s teeth to become strong. Another believed that each child receives calcium from her mother and that was the reason she had healthier teeth than her younger siblings. She explained that as the first-born child she was lucky to receive all calcium needed. Further, some women mentioned the sun as the source of oral health. A woman referred to the elders and the general belief in her culture when she said:

‘The sun makes so many vitamins which are good for the teeth’

Another also referred to the common knowledge that circulated in her surroundings since she was a child. She said:

‘All problems with the gums and all problems with the teeth are… I believe… they start because of poor hygiene’

### Needs-related purpose of seeking information

This category contains the reasons which emerged in the interview data, for seeking information: *dental emergency, need for clarification* and *teaching tool*. The information seeking was carried out due to the emerging needs and depended on urgency of the condition, situational context, and available resources. Emerged needs motivated participants to seek information and take necessary actions to solve them. Participants believed that emerged needs could be mitigated with proper information.

#### Dental emergency

The mothers stated that one reason for seeking information was a child’s dental problem. They reported searching the internet in cases of minor issues or a common question, such as an exfoliating primary tooth or the yellowish colour of newly erupted permanent teeth. A number of mothers stated that unlike the Swedish recall system for children, dental services in the home country were contacted only in case of a dental emergency or an observed dental problem, such as this mother:

‘In my home country we don’t have such a dental system where as a child you go to the dentist for a check-up every year. If you have a hole or if you lose a tooth or have to have teeth filled, then you go to the dentist but not otherwise’

#### Need for clarification

Some women stated that dental professionals provide all the information they needed and searching for further information is necessary only to refresh one’s memory, while others perceived that the information provided may be inadequate for different reasons and additional information is needed. For example:

‘Sometimes the dentist doesn’t explain thoroughly enough, or I don’t understand and he hasn’t the time: ‘ok I have to run’ or he doesn’t speak my language. I can’t ask. I can’t ask as easily as I can (in my own language)’

Also mentioned was the importance of complying with professional recommendations to seek clarifying information. A woman explained that one is supposed to listen to the advice of professionals when they recommend googling particular information or visiting a particular webpage for detailed information. Another commented that professionals cannot explain everything at one visit. They just give some advice and refer one to an information page in the informant’s native language. She said:

‘They just can’t explain the whole. They give a little advice and then there might be a page in my native language: Read that thoroughly!’

#### Teaching tool

Some mothers stated that they obtained oral health information for use as a teaching tool for their children. A mother mentioned that together with her children she read a book about toothbrushing, while another described using the internet as an educational tool for her children. She said:

‘If I am to show my children pictures of how important it is for them to brush their teeth’.

### Trustworthiness assessment

This category contains the essence of how the participants assess the credibility of oral health information. They placed value on the source of information, invested effort in critical assessment of the information being presented to them and focused on resources which they thought were authentic to receive oral health-related information. There are three sub-categories: *professional information, majority opinion* and *recurring information*.

#### Professional information

The participants described relying on the dental clinicians’ professional qualifications, such as this woman, who spoke about dental professionals as a trustworthy source of information:

‘They are the ones who have studied and trained and worked in the profession for several years and that is why one has to rely on them, they are knowledgeable’

Another stated that she relied on receiving appropriate information when needed and that dental professionals know what she needs to know. She said:

‘I don’t know. People like you know what I need to know’

Some mothers spoke about comparing information from different sources. A mother said that one cannot rely just on information on the internet; it needs to be compared with professional advice. She stated:

‘Sometimes google lies’

Another considered the information on the internet to be impersonal and therefore not credible. She expressed it this way:

‘I don’t know, but one should not rely so much on Google because it isn’t a dentist. I don’t meet the dentist face to face’

Some participants believed that the best way to obtain information is to focus on one source of information, such as this woman:

‘I think it’s good if you can concentrate just on the Public Dental Service’

#### Majority opinion

Several participants stated that they consider majority opinion when seeking oral health information. One woman, for example, chose to contact a particular dentist because there were many who recommended him. Another shared her strategy for credibility control of information on the internet:

‘I read many pages then I look at which pages lots of people read and which pages I am told are good’

#### Recurrent information

The participants perceived that information is more trustworthy if it recurs in different sources and if it is about common issues. A mother shared her thoughts about information on the internet:

‘If there is any correct information then the same information appears on many sites. So that’s why I rely on them, it is the same information’

Another stated that information about common issues, such as the importance of toothbrushing, does not necessarily have to come from professionals but it is sufficiently credible if many say the same thing.

## Discussion

This qualitative study explored OHL in a purposive sample of migrant mothers, with special reference to sources they used and their reasons for seeking oral health information and also how they determined the credibility of the information they obtained. The main finding of this study was that migrant mothers used information sourcing as a pathway to solve oral health literacy tasks.

### Strengths and limitations

One limitation of this study could be the small number of participants. However, applying the *information power model* in the planning and during data collection facilitated assessment of the adequacy of our final sample size [15]. We considered a number of criteria. The public dental care coordinators helped to involve local agencies working with migrant parents, which in turn helped to select participants with characteristics specific for the study. Dense sample specificity enhanced information power: thus fewer participants were needed. The interviewer is experienced in qualitative research and has long experience of interviewing in her professional role: she has the ability to inspire confidence and establish a good dialogue. She is also experienced in meeting migrant families in the dental care context. Moreover, in the planning phase, interviews were conducted with public dental health coordinators for migrants and with the cultural interpreters representing each language community, with the aim of enhancing the interviewer´s understanding of migrant families’ oral health issues. These facts show that the interviewer has more than average background knowledge about the subject of this study, which can indicate a strong quality of dialogue and the need for fewer participants. Furthermore, our study uses analysis of in-depth interviews from selected participants, which also justifies the small number of participants. Although the study is not theoretically grounded, which might indicate a need for more participants, the authors are experienced in empirical matters of OHL. The aim of the study and research questions are neither broad nor narrow. Considering all of the above and our continuous assessment of information power during the planning and data collection, we could retrieve solid data from seven strong quality interviews.

Initially, we intended to interview migrant parents for this study. However, only mothers expressed an interest in participating. This could be because in the study participants’ countries of origin, it is traditionally the mothers who are responsible for children’s diet and oral hygiene habits.

According to the literature, choosing participants with diverse experience improves the potential to shed light on the research question from a variety of aspects [12,14]. Our participants were similar in that they all were women with small children and had arrived in Sweden at around the same time. On the other hand, the participants’ different ages, different numbers of children, different countries of origin and different social background, such as social class, educational level and working life experience, contributed to a richer variation of the phenomena. The fact that the study was undertaken in a Swedish context could influence the participants’ OHL related to that context. Therefore, the OHL of our participants could differ from that of migrant parents in other contexts.

All interviews and transcriptions were conducted by the first author, and this could influence the interview conditions and the analysis. However, the interviews, the coding and the categorization were discussed among the authors and the SANT-network until consensus was achieved. Quotations from the interview were also included to help readers make their own judgment. Krippendorff (2018) argues that a text never implies one single meaning, just the most probable meaning from a particular perspective [17]. Thus, our interpretation should be considered as one possible interpretation of migrant parents’ information sources, their reasons for seeking information and their assessment of the credibility of the oral health information obtained.

All interviews were conducted and transcribed in a relatively short period of time and all the interviews followed the interview guide. These factors reduce the risk of inconsistency in data collection [12]. For interpretation of accounts from the participants, we used the research questions as a basis for the analysis. This helps in addressing challenges to dependability, allowing for systematic differentiation between categories [18].

### Findings

It has been suggested that in order to address health disparities in migrant populations, we should focus on sources of health information, because access to such information is crucial to people making health decisions on behalf of their children [19]. Our findings are in accordance with previous studies conducted in migrant populations, disclosing that migrant mothers utilized sources of oral health information that were accessible and convenient, such as family members, dental professionals and the Internet [20–22]. Moreover, the participants’ OHL was influenced by social contexts in the places where they grew up and lived, such as schools, neighbourhoods, and the environment in the host country. However, the findings indicated that for the majority of migrant mothers in this study, the internet was a major source of oral health information. One reason might be the widespread internet access and high digitalization in Sweden. Increasing accessibility and penetration of internet services have greatly influenced how people gather health-related information. Another reason might be that because the informants were young (median age was 30 years), they may have been active and adaptable in using the internet for information seeking. However, the internet was mainly used for seeking information on minor oral health issues or common questions. In case of more serious oral health issues or a dental emergency, dental professionals were the primary source of information for our participants. These findings suggest that dental professionals are seen by migrant mothers as an obvious source of authentic oral health related information.

In the current study, the main reason for seeking information was in case of a dental emergency which entailed subsequent appointments for dental treatment. It has previously been reported that migrant women and their children access dental services primarily for emergency care rather than for preventive care [23]. In many countries, preventive oral health care is not part of routine healthcare as it is in Sweden, and this can impact attitudes as to when to seek care. In Sweden, the regional health authorities have a special responsibility for promoting oral health among children and young people [24]. Oral health care is free of charge for all individuals up to 23 years of age. Regular check-ups and measures for prevention of oral diseases cover practically all children and adolescents. Information, oral hygiene instruction, dietary recommendations and fluoride should be part of regular recall visits [25]. However, migrants arriving from countries with a different oral health care system might not be aware that these benefits are available for their children. Previous studies have shown that immigrants experienced the need for information about the health care system and about their rights in health issues [26,27]. Riggs and colleagues in a review of refugee child oral health [28], highlighted challenges that families face when accessing dental services in their new country. Moreover, inadequate knowledge of the language is perceived as a barrier to communication with health care professionals [26,27]. Our findings also indicated not only language barriers but also a need for clarification of information received from dental professionals. Therefore, to improve OHL in migrant parents, oral health care delivery should be culturally more appropriate, including using the native language and information channels available to migrant parents.

It should be noted that the study participants comprised only mothers. Some previous studies have found correlations between health literacy and gender [29–32]. However, the results of these studies are inconsistent. It is unclear whether gender affects health literacy.

A novel finding was that our participants were looking for pictorial support on the internet to use it as an oral health teaching tool for their children. These findings suggest that migrant mothers believe that pictorial support can promote oral health literacy in their children. It has previously been shown that pictorial support used by health professionals facilitated communication with migrants and that the health professionals were motivated to use it and perceived a need for it [33]. Thus, in preventive dental practice, pictorial support might be a valuable tool for facilitating communication with migrant parents and their children. Pictorial information about oral health in their native language, developed by professionals, could be provided to migrant parents for use at home as a teaching tool for their children.

It has been suggested that obtaining health information goes beyond the process of merely acquiring knowledge. Although the migrant mothers in this study frequently used the internet as a primary source of oral health information, they did not always regard it as the most reliable information channel. Our findings revealed that the participants utilized multiple sources of information simultaneously. They verified the information they received from one source with another, such as confirming information gathered on the Internet or from friends, with that provided by dental professionals. In accordance with a previous study [26], our findings suggest that to improve OHL in migrant mothers, dissemination of oral health information needs to embrace a variety of different channels outside the dental care system. Moreover, the information should be consistent and recurrent.

Furthermore, Healthy People 2030 and its updated definition of health literacy, which now includes both personal and organisational components, encourages health professionals and health care leaders to take a systemic approach to improving health literacy for all [34]. Healthy People 2030 acknowledges the level of complexity of health-related information and therefore highlights the responsibility of organizations at any level to make information and services easy to find, understand and use. Our findings showed that migrant mothers’ sources of and approaches to obtaining oral health-related information include several channels and strategies. These can be used by all institutions working with migrant families in collaboration with the dental care organizations to help improve OHL in migrants.

Additionally, our findings can be accommodated within the OHL framework and particularly withing the culture and society and the health system domains [35]. Participants’ OHL exists within the context of culture and society and within the interaction that they have with the dental care system, and in that way complements their children’s oral health.

## Conclusions

This study indicates that migrant mothers of young children wholeheartedly trust the source of information used. In seeking information about oral health, they mainly use accessible information sources such as family, the internet or dental professionals. The main reason for seeking information was a dental emergency, followed by the need for clarification of information received from professionals at dental appointments and as a teaching tool to educate their children in oral health. The migrant mothers perceived oral health information to be most reliable when delivered by dental professionals, when it was found to recur in different sources or constituted majority opinion. The findings suggest that OHL in migrant mothers of young children may be improved if (1) the dissemination of oral health information embraces channels accessible to migrant mothers, (2) pictorial support is used in communication, (3) dental care is adapted to be culturally more appropriate, and (4) oral health information is consistent, recurring in several sources.
